# The Physicians’ Mutual Benefit Association of Texas

**Published:** 1888-05

**Authors:** 


### THE PHYSICIANS' MUTUAL BENEFIT ASSOCIATION OF TEXAS.

---

Pursuant to notice a number of members of the Physicians' Mutual Benefit Association of Texas met in the parlor of Harmony Hall, Galveston, April 26th, and Dr. R. H. Harrison, Sr., of Columbus, was called to the chair. The Secretary, Dr. Daniel, made a report of the status and the workings of the order to date.

The Constitution and By-Laws were read, but no alterations were made or proposed.

The original Board of Directors having all lost their membership, a new board was elected, consisting of Drs. Swearingen and McLauglin, Austin; Dr. Cupples of San Antonio; Dr. W. P. Burts of Fort Worth; Dr. J. K. Stone of Richmond; Dr. Hewson, Orange, and Dr. T. J. Bell, of Tyler. Dr. F. R. Martin, of Kyle, and M. A. Taylor, of Austin, were elected Vice-Presidents. On motion, an executive board was appointed, consisting of the President and Vice-Presidents and Secretary with two of the Directors, to-wit: Drs. Swearingen and McLaughlin. This Board is to have general charge of the business, and to act for the Association in matters requiring prompt attention where there would be delay in getting the Directors assembled.

On motion of Vice-President Martin, the Board of Directors was authorized to issue a circular letter to the profession of Texas, setting forth the aims, objects and scope of the organization, and urging its claims upon their attention. This will be done at the earliest practicable moment.

It is hoped and expected that this step will awaken a renewed interest in this truly benevolent organization and inspire confidence, not only in its integrity, but in the permanency of its success. Physicians, who have held aloof from sheer indifference, will see now that it is in the hands of prominent, leading physicians, representing all sections of the State, who are determined to push it; and instead of the pittance of the hundred odd dollars, or so, heretofore contributed to the families of deceased brothers we hope to see it mount up into the thousands.

It was thought best not to increase the death assessment, nor the annual dues; both remain at the nominal sum of $1, and instead, it was determined to increase the number of members.

---

The Lone Star Medical Association (Colored), held its second annual session in Austin, May 14th and 15th. J. F. McKinley, M. D., President; Dr. Majors, Secretary. There was a good attendance, considering the small number of colored physicians in the State and their meeting was characterized by order and a sincere desire to advance the cause of medicine. We acknowledge the courtesy of an invitation and were present, listening to the proceedings with much interest. They meet next year at Houston.

